# High-throughput genotyping of wheat-barley amphiploids utilising diversity array technology (DArT)

**DOI:** 10.1186/1471-2229-13-87

**Published:** 2013-06-03

**Authors:** Almudena Castillo, María C Ramírez, Azahara C Martín, Andrzej Kilian, Antonio Martín, Sergio G Atienza

**Affiliations:** 1 Instituto de Agricultura Sostenible, IAS-CSIC, Apdo. 4084, Córdoba E-14080, Spain; 2 Diversity Arrays Technology Pty Ltd, PO Box 7141, Yarralumla, ACT 2600, Australia

**Keywords:** Tritordeum, DArT, Chromosome substitution, GISH, EST, SSR markers

## Abstract

**Background:**

*Hordeum chilense*, a native South American diploid wild barley, is one of the species of the genus *Hordeum* with a high potential for cereal breeding purposes, given its high crossability with other members of the Triticeae tribe. Hexaploid tritordeum (×*Tritordeum* Ascherson et Graebner, 2n=6×=42, AABBH^ch^H^ch^) is the fertile amphiploid obtained after chromosome doubling of hybrids between *Hordeum chilense* and durum wheat. Approaches used in the improvement of this crop have included crosses with hexaploid wheat to promote D/H^ch^ chromosome substitutions. While this approach has been successful as was the case with triticale, it has also complicated the genetic composition of the breeding materials. Until now tritordeum lines were analyzed based on molecular cytogenetic techniques and screening with a small set of DNA markers. However, the recent development of DArT markers in *H. chilense* offers new possibilities to screen large number of accessions more efficiently.

**Results:**

Here, we have applied DArT markers to genotype composition in forty-six accessions of hexaploid tritordeum originating from different stages of tritordeum breeding program and to *H. chilense*-wheat chromosome addition lines to allow their physical mapping. Diversity analyses were conducted including dendrogram construction, principal component analysis and structure inference. Euploid and substituted tritordeums were clearly discriminated independently of the method used. However, dendrogram and Structure analyses allowed the clearest discrimination among substituted tritordeums. The physically mapped markers allowed identifying these groups as substituted tritordeums carrying the following disomic substitutions (DS): DS1D (1H^ch^), DS2D (2H^ch^), DS5D (5H^ch^), DS6D (6H^ch^) and the double substitution DS2D (2H^ch^), DS5D (5H^ch^). These results were validated using chromosome specific EST and SSR markers and GISH analysis.

**Conclusion:**

In conclusion, DArT markers have proved to be very useful to detect chromosome substitutions in the tritordeum breeding program and thus they are expected to be equally useful to detect translocations both in the tritordeum breeding program and in the transference of *H. chilense* genetic material in wheat breeding programs.

## Background

Interspecific and intergeneric hybridization is a useful tool in the breeding of cultivated species of Triticeae tribe. This technique has been widely used to transfer desirable traits from wild to cultivated species [1-3] and to increase the genetic variation of the species by developing new synthetic hexaploid wheats [4-6] or using *Triticum urartu* (donor of the A genome) for durum wheat breeding [7]. Intergeneric hybrids between *Hordeum* and *Triticum* genus were attempted since the beginning of the 20^th^ century. Since the first hybrids reported by [8] numerous hybrid combinations between both genera have been produced and reviewed by [9]. However, only a few fertile hybrids have been obtained by chromosome doubling with colchicine. These were the hybrids between *T. timopheevii* × *H. bogdanii*[10], *H. chilense* × *T. aestivum*[11] and *H. chilense* × *T. durum*[12]. Among *Hordeum* species, *Hordeum chilense* Roem. et Schultz. is a native South American diploid perennial wild barley (2n = 2× = 14), included in the section Anisolepsis [13]. It belongs to a heterogeneous group of South American *Hordeum* species and it is one of the species of the genus *Hordeum* with a high potential for cereal breeding purposes, given its high crossability with other members of the Triticeae tribe and other interesting characteristics [14]. In recent years, molecular and cytological techniques have been developed in *H. chilense* for basic cytogenetic research, genetic diversity studies and monitoring *H. chilense* chromosomes in wheat genetic background. Several types of molecular markers, including Random Amplified Polymorphic DNA (RAPD) [15], Sequence Characterized Amplified Regions (SCARs) [16], Cleaved Amplified Polymorphisms (CAPs) [17] or DArTs [18] have been developed *de novo* for *H. chilense*. Similarly other markers have been transferred from wheat and barley species including Sequence Tagged Sites (STSs) [19], genomic or EST-derived simple sequence repeat (SSRs) [20,21], ESTs [22-24] or Conserved Orthologus Sequences (COS) [18].

In addition to marker-assisted selection, genomic in situ hybridization (GISH) and fluorescence in situ hybridization (FISH) are useful tools which have been used for identifying *H. chilense* chromosomes (or chromosome translocation) in wheat background [25-27].

Tritordeums (×*Tritordeum* Ascherson et Graebner) are the fertile amphiploids obtained after chromosome doubling of hybrids between wheat (*Triticum* sp.) and *H. chilense*. They have been synthesised at different ploidy levels and genome constitutions, of which hexaploids tritordeums (2n = 6× = 42, AABBH^ch^H^ch^) have been subject of breeding program [28]. The favourable agronomic traits shown by tritordeums, such as high biomass yield, number of spikelets/spike, seed size and high protein content, suggested its potential to become a new crop [29] which was confirmed nearly two decades later [30]. The high seed carotenoid content [31,32] of this species constitutes also an interesting characteristic in the context of developing functional foods. Accordingly, tritordeum has received attention as a potential crop in the last years and a breeding program has been developed including the development of chromosome substitutions involving D and H^ch^ genomes. While the chromosome substitution program has obtained successful results for free threshing ability [33] or improved bread-making quality [34], it has also complicated the genetic composition of breeding materials since different chromosome substitutions may be present involving D and H^ch^ genomes. Until now tritordeum lines were inspected for *H. chilense*/wheat substitutions using physically mapped markers per chromosome [33] and using cytogenetics tools like GISH [22,24] and FISH [35]. While cytogenetics is useful for a limited set of lines, it is impractical for large amounts of entries included in a breeding program. Furthermore, the use of a limited number of molecular markers is not adequate for managing a large collection of germplasm since the rate of error in classification may be too high compared to whole genome profiling.

Based on the above considerations we embarked on development of Diversity Arrays Technology (DArT) since this technology can overcome these constraints. In addition DArT technology offers low cost per data, high throughput and sequence-independent genotyping [36,37] and allows simultaneously determination of several hundred to several thousands of polymorphic loci spread over the genome [38]. The high number of DArT markers generated in a single assay not only provides a precise estimate of genetic relationships among genotypes, but also their distribution over the genome offers real advantages for a range of molecular breeding and genomics applications.

DArT markers have been developed in more than 70 species (http://www.diversityarrays.com), including cereals such as cultivated barley (*Hordeum vulgare*), wheat (*Triticum aestivum*), durum wheat (*Triticum durum*), and recently wild barley (*H. chilense*).

DArT array have also been proved useful for polyploidy species with highly complex genomes [39]; to provide a fast and accurate means of determining the extent of introgression of the genome of the diverse parent in interespecific hybrids [40] or to evaluate progenies derived from interespecific crosses between *T. aestivum* and *T. durum*[41].

This paper evaluates the effectiveness of DArT as a high-throughput genotyping technology in tritordeum. This technology offers tritordeum breeding program an alternative approach to whole-genome profiling providing high quality markers that can be exploited in a range of molecular breeding and genomics applications in tritordeum. Additionally we set out to explore the possibility of using DArT array for genome background screening in tritordeum and to determine terms of discriminative ability using 1RS/1BL translocation lines.

## Results

### DArT array composition

A total of 2,372 out of 11,000 new DArT clones generated previously from *H. chilense*[18] were printed in the array together with clones derived from other species developed in parallel projects (2,071 hexaploid wheat-derived markers, 290 from *H. vulgare*, 208 from triticale and rye). From 4,941 clones identified on the array, a set of 3,357 markers were selected following the quality criteria explained above (P value and reproducibility), with 2,377 polymorphic markers used for diversity analyses of the 46 tritordeum lines (Additional file 1).

Wheat-*H. chilense* chromosome addition lines allowed the physical location of a total of 2,209 *H. chilense*-derived markers. Out of these, 1,280 markers could be assigned to a specific *H. chilense* chromosome on basis of two replicates (Additional file 2). The remainder could not be assigned because a) they were also present in genomic representations generated from wheat; b) they were absent in representations generated from H1, the accession used to develop *H. chilense*-wheat addition lines; c) they were found in H1 and absent in all the addition lines which suggests that these markers are located in either 2H^ch^L or 3H^ch^, since addition lines carrying these chromosomes are not available or d) because they gave signal in two chromosomes and they were excluded of the study.

A subset of 450 DArT markers was shared with our mapping project [18], where Diversity Arrays technology (DArT) genomic libraries were developed from *H. chilense* accessions. Out of these, 378 physically mapped to a specific *H. chilense* chromosome matched the quality criteria in the present work.

### Genetic analysis

PIC ranged from 0.04 to 0.5, with an average of 0.31, for a bi-allelic marker, the minimum and maximum PIC values are 0 and 0.5, respectively. Two analyses were performed. The first of them included all the DArT markers while the second used polymorphic markers derived from *H. chilense* and D-genome, since we were looking for H^ch^/D chromosomes substitutions. The latter analyse produced a better discrimination among tritordeum lines and thus only results obtained using this subset of markers are shown. Brieftly, both the dendrogram and the PCoA analyses obtained with all markers included two non-substituted tritordeums with the substituted ones. Besides, the structure analysis separated between the substituted tritordeums and non-substituted ones. However, further analysis was required to differentiate among the different substitions and it was unable to distinguish the DS6D (6H^ch^) and other substitutions.

**Figure 1:**
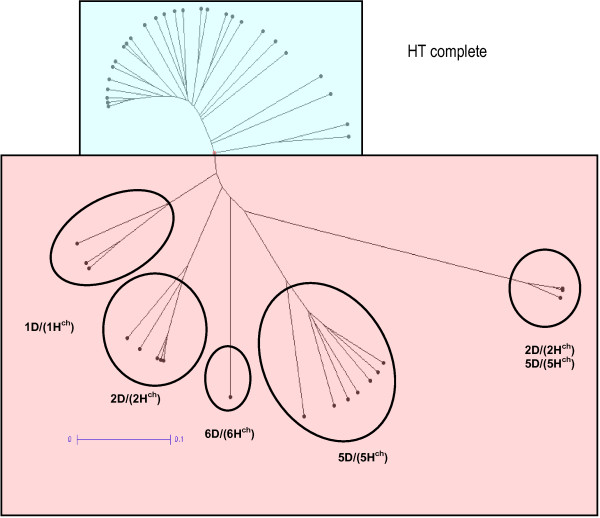
**UPGMA dendrogram (shown as Radial tree).** UPGMA dendrogram based on 1,145 *H. chilense* and D-genome polymorphic DArT markers using Jaccard’s similarity matrix, showing the relationship between 46 tritordeum lines. Scale bar represent the genetic distance as determined using the Jaccard coefficient.

First, the 1,145 polymorphic markers derived from *H. chilense* and wheat D genome were considered to construct a dendrogram (Figure 1). The accessions were separated into two major clusters with a cophenetic correlation value of 0.912 indicating an excellent fit of the similarity matrix data to the tree topology. The first group was formed by 27 accessions and it included those tritordeums with complete chromosome composition and the second group clustered 19 accessions carrying suspected or known chromosomal substitutions. Within this latter group, the inspection of the DArT markers dataset allowed to assign each subgroup to a different chromosome substitution. Five subgroups were differentiated, four of them comprised a single chromosome substitution (DS1D (1H^ch^), DS2D (2H^ch^), DS5D (5H^ch^) and DS6D (6H^ch^)) and one group had three genotypes with double chromosome substitution (DS2D (2H^ch^) and DS5D (5H^ch^)) (Figure 1).

**Figure 2:**
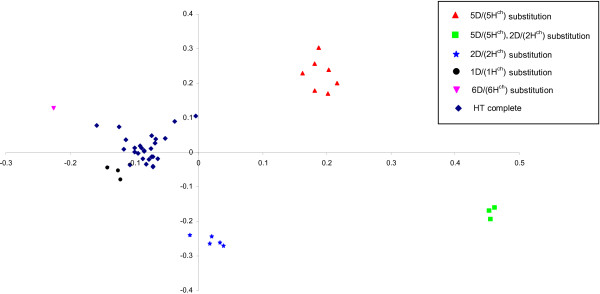
**Principal coordinate analysis of 46 tritordeum lines based on the *H. chilense *and D-genome polymorphic DArT markers.** The diagrams show the positions of individual accessions in the space spanned by the first two coordinates. The genetic composition of the lines is shown as follows: triangle: DS 5D (5H^ch^); square: double DS2D (2H^ch^) and 5D (5H^ch^); star: DS2D (2H^ch^); circle: DS1D (1H^ch^); inverted triangle: DS 6D (6H^ch^) and diamond: euploid tritordeum.

The principal coordinate (PCoA) was also constructed based on genotype data from the polymorphic DArT markers derived from *H. chilense* and wheat D genome. A two dimensional scatter plot of the 46 tritordeum genotypes, shown in Figure 2, confirmed the five different subgroups found by UPGMA dendrogram although the DS6D (6H^ch^) and the DS1D (1H^ch^) substitution were placed near the non-substituted tritordeums. The two first dimensions of PCoA explained 34% and 25% respectively of the variation present in the genetic distance calculated between genotypes. Again, tritordeums carrying D/H^ch^ substitutions were clearly differentiated from non-substituted tritordeums (Figure 2).

**Figure 3:**
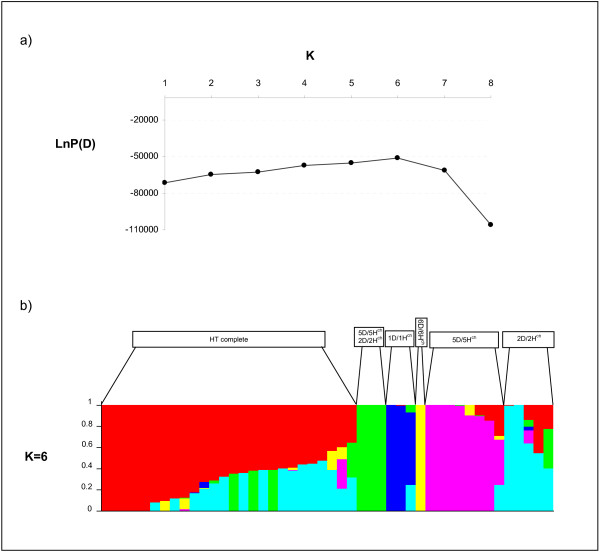
**Membership of tritordeum lines using DArT markers and the package STRUCTURE. a**) Evolution of the natural log probability of the data, which is proportional to the posterior probability of K, against K (number of populations) **b **) Each individual is represented by a line partitioned in five coloured segments that represent the individual’s estimated membership fractions to each one of the six clusters.

Genetic structure analysis detected an underlying structure, with six groups, based on the criterion of maximization of the natural log probability of the data, which is proportional to the posterior probability of K [42]. The results pointed out to a clear cut-off point for the number of groups in our sample for K=6 (Figure 3a). The genotypes were spread among the six groups as follows: Group I comprised 27 genotypes, including all tritordeums with complete chromosome composition, Group II includes three accessions from double chromosome substitution DS5D (5H^ch^) and DS2D (2H^ch^), Group III comprised three genotypes from DS1D (1H^ch^) chromosome substitution, Group IV included one line from DS6D (6Hch) substitution, Group V had seven genotypes from the DS5D (5H^ch^) chromosome substitution and group VI included five lines from the DS2D (2H^ch^) chromosome substitution (Figure 3b).

**Figure 4:**
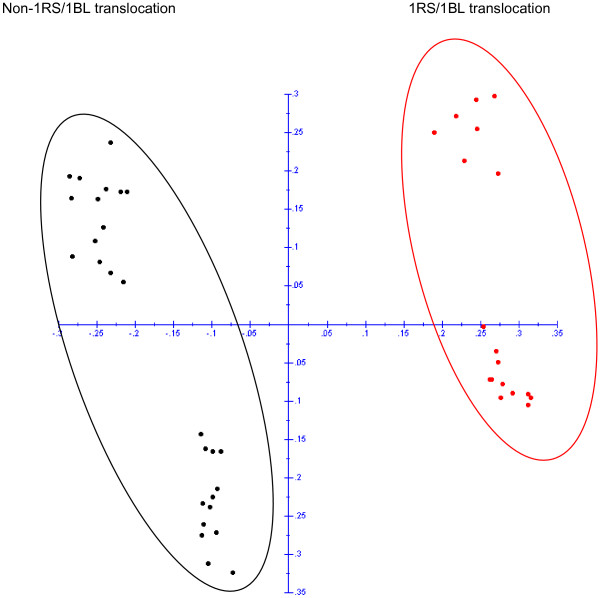
Principal coordinate analysis of 46 tritordeum lines based on all the rye and triticale polymorphic DArT markers regardless their chromosome position.

The potential of DArT markers to detect translocations was studied on the basis of 114 polymorphic DArTs markers derived from rye and Triticale. PCoA analysis of these markers which revealed a clear discrimination between 1RS/1BL and non-1RS/1BL translocation lines (Figure 4). Once we confirmed the 1RS/1BL translocation with the specific primers for the locus SEC-1b, we inspected the DArT data file to identify markers discriminating the eighteen tritordeum lines carrying the 1RS/1BL translocation. Thirty three markers were only present in the tritordeums with the translocation 1RS/1BL (Additional file 3). Most of them (31) were derived from rye or triticale and out of these; twenty eight were previously assigned to chromosome 1B and three not assigned to any chromosome. In addition, a single marker from each wheat (1B) and barley (1H) also discriminated between translocated and non-translocated lines. Similarly, chromosome specific EST and SSR specific markers (Table 1) were used to verify the chromosome composition of the different lines studied. These markers either produce different amplification products in *H. chilense* and wheat or exclusively amplify the *H. chilense* genome, being therefore useful to detect chromosome substitutions.

**Table 1:** Description of PCR-based markers used for the characterization of tritordeum lines

**a)**	
EST-markers (Nasuda et al. [24])	Chromosomal location
k01339	1H^ch^S
k01437	1H^ch^L
k04721	2H^ch^S
k00579	2H^ch^L
k02539	4H^ch^S
k04725	4H^ch^L
k01323	5H^ch^S
k04947	5H^ch^L
k01062	6H^ch^S
k01193	6H^ch^L
k04783	7H^ch^S
k04058	7H^ch^L
**b)**	
SSR-markers (Röder et al. [53])	Chromosomal location
Xgwm337	1D
Xgwm261	2D
Xgwm161	3D
Xgwm194	4D
Xgwm272	5D
Xgwm325	6D
Xgwm44	7D

a) Barley EST-SSR markers previously assigned to *H. chilense* chromosomes, b) D-genome specific SSR markers.

### Cytogenetic characterization by GISH

**Figure 5:**
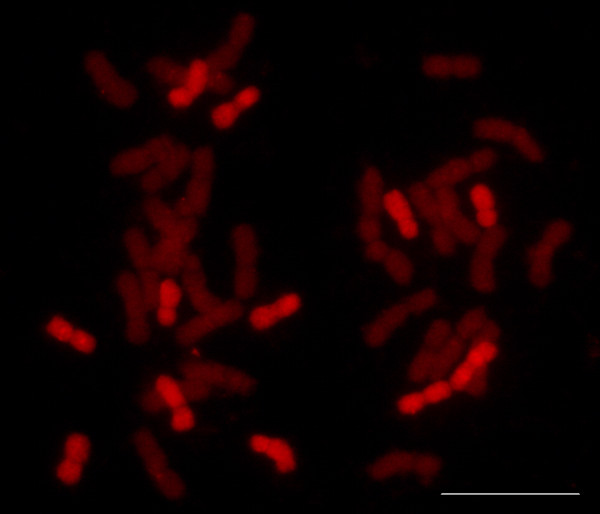
**GISH using *H. chilense *genomic DNA as probe (detected with biotin and shown in red) in metaphase cell of a chromosome substitution line.** The line carries 12 *H. chilense* chromosomes suggesting a single chromosome substitution. Scale bar equal 10 μm.

Genomic in situ hybridization was used to assess the chromosome substitutions using *H. chilense* genomic DNA as probe (detected with biotin). Sixteen single chromosome substitution lines were found to have only 12 chromosomes from *H. chilense* corresponding with DS1D (1H^ch^), DS2D (2H^ch^), DS5D (5H^ch^) and DS6D (6H^ch^) while three double chromosome substitution lines corresponding to DS2D (2H^ch^) and DS5D (5H^ch^) showed 10 chromosomes from *H. chilense* (Figure 5). The rest of the lines were unsubstituted tritordeums carrying all fourteen *H. chilense* chromosomes. In addition, eighteen lines showing 1RS/1BL translocations were detected using *S. cereale* genomic DNA as probe (data not shown).

## Discussion

The availability of reliable molecular markers is of great importance for plant breeding. The ideal molecular marker technique should generate hundreds of molecular markers that cover the entire genome in a single, simple and reliable experiment [43]. Genome-wide molecular markers are used for germplasm characterization, assessment of genetic diversity, to accelerate introgression or backcrossing programs, and for the mapping of complex traits. The high number of DArT markers generated in a single assay covering the whole genome allows the screening of a large number of genotypes in a rapid and efficient way.

Previous characterizations of the tritordeum have relied on cytogenetics using GISH and PCR-based markers [33,34]. The GISH technique is laborious and unsuitable for high-throughput analyses. Similarly, the use of small sets of molecular markers may fail to detect translocations between H^ch^ and D genomes in a high proportion and thus they only partially fulfil the requirements of our research programs. The development of DArT markers in *H. chilense* has facilitated the use of these markers in tritordeum breeding program and related projects. In this work, we took advantage of the existence of a large number of DArT markers previously developed from hexaploid wheat, *H. vulgare*, Triticale, *S. cereale* and *H. chilense*. A high percentage of DArT markers (48%) were polymorphic among the accessions studied which is higher than observed for DArT in other crops. For example a polymorphism rate of 9.4% observed in wheat [39] while the polymorphism rate for DArT markers originating from hexaploid wheat, triticale, and rye as 8.6%, 23.4% and 23.8%, respectively in triticale [44]. Since the DArT array was designed to maximize polymorphism rate considering previous experiments, this has resulted in a much higher polymorphism rate than reported in other crops. The average PIC value of 0.31 found in the current study was informative and comparable to values previously observed for barley (0.38) [38], wheat (0.31) [39], hop (0.34) [45], and triticale (0.37) [44].

*H. chilense* and D-genome polymorphic markers were selected for genetic analysis to increase the power of detection of chromosome substitutions involving these genomes.

All three diversity analyses showed a good level of consistence to discriminate between substituted and non-substituted tritordeums. Both the dendrogram and the structure analysis grouped separately each type of chromosome substitutions but the first is much faster. Finally, the PCoA analysis discriminated well among groups. However, the DS6D (6H^ch^) and the DS1D (1H^ch^) groups appeared closely to non-substituted tritordeums which might lead to errors in classification in future analyses. In any case, we think that the application of DArT to the tritordeum breeding program would benefit from the analysis with all three methodologies since lines grouped differently in different methodologies may require a deeper analysis of the results.

The development of substitution lines has proven to be an efficient tool for the improvement of important agronomic traits. For instance, a major contribution to triticale improvement was achieved by the use of ‘Armadillo’, a line showing improved fertility due to the chromosome substitution DS2D (2R) [46]. Although subsequent international yield trials indicated that complete hexaploid triticales showed a better agronomic performance than substitutes [47], the contribution of Armadillo to triticale improvement was very important. Similarly, D/(H^ch^) substitutions have allowed the improvement of breadmaking quality [34] or free-threshing ability [33] but it is expected that tritordeums without chromosome substitutions will show superior performance over substituted ones in the future, as was observed in triticale. Nevertheless, the occurrence of translocations involving H^ch^ and D chromosomes may still add interesting characteristics, as seen in wheat. Indeed, the 1RS/1BL translocation is one of the most frequently used alien introgresions in wheat-breeding programs throughout the world [48]. The 1RS/1BL has positive effect on agronomic traits like yield performance, however, 1RS carries the Sec-1 locus coding for ϵ-secalin, which results in negative effects on bread-making quality. DArT markers clearly discriminated the lines with the 1RS/1BL translocation and thus, it is expectable that they will also allow the detection of D/(H^ch^) translocations when available. Accordingly these markers will prove very useful in parallel ongoing projects such as the development of hybrid wheat [49-51].

## Conclusions

In conclusion, DArT markers allowed discrimination of the substitution lines involving the D genomes/H^ch^. All three methodologies clearly separated complete from substituted tritordeums but the combination between them will allow better discrimination of the specific lines. DArT markers also allowed the detection of translocations as evidenced from by the study of the 1RS/1BL translocation and, thus, they will very useful both in the tritordeum breeding program and the common wheat hybrid system.

## Methods

### Plant material

Forty-six accessions of hexaploid tritordeum originating from different stages of tritordeum breeding program were evaluated. *Triticum aestivum* cv. Chinese Spring (CS)-*H. chilense* addition lines for complete chromosomes 1H^ch^, 4H^ch^, 5H^ch^, 6H^ch^ and 7H^ch^ (named CS MA 1H^ch^-1H^ch^S, CS DA4H^ch^, CS DA5H^ch^, CS DA6H^ch^ and CS DA7H^ch^, respectively, where MA refers to monosomic addition and DA means disomic addition), and the wheat-(CS)-*H. chilense* ditelosomic addition lines CS DA1H^ch^S, CS DA2H^ch^S, CS DA5H^ch^L, CS DA6H^ch^S, CS DA7H^ch^α, CS DA7H^ch^β were used to assign markers to specific chromosomes. *T. aestivum* cv ‘Chinese Spring’ and *H. chilense* accession H1 were also included. DNA was extracted from young leaf tissue from a single plant of each genotype using the protocol recommended by Triticarte Pty. Ltd. (http://www.triticarte.com.au).

### Genotyping

A total of 4,941 DArT clones were printed in the array. Most of them were derived from *H. chilense* (2,372) and hexaploid wheat (2,071). The array was completed with markers from barley (290) and rye and triticale (208). The resulting composite array was then used to fingerprint tritordeum and addition lines using the standard DArT protocol [52]. Only DArT markers with a quality criteria, P value and reproducibility higher than 80 and 97% respectively, were selected.

Polymorphism information content (PIC), a measure of the informativeness of a genetic marker, was also calculated for each marker as follows; PIC = 1 − *Σ*Pi^2^, where Pi is the frequency of the *i*th allele in the examined genotypes. Chromosome substitutions in tritordeum were verified as previously described [33]. A set of Expressed sequence tagged (EST) markers [23,24] and wheat chromosome-specific SSRs [53] were used to verify the presence of *H. chilense* and wheat chromosomes respectively (Table 1). 1RS/1BL translocations were detected using the primer pair SecA2/SecA3 designed specifically to amplify a sequence of ω-secalin gene (locus SEC-1b) located on the short arm of the rye chromosome 1R [54].

### Data analysis

The data matrix containing the 0/1 scores of the *H. chilense* and D-genome-derived polymorphic DArT markers was transformed to a genetic distance matrix using Jaccard’s coefficient [55]. The genetic distance matrix was used to produce an unrooted Unweighted Pair Group Method with Algorithmic Mean (UPGMA) dendrogram using the program Dissimilarity Analysis and Representation for Windows, also known as DARwin [56]. Also, the first two principal coordinates of the resulting Jaccard matrix were extracted to display the position of the accessions in a two-dimensional space (as an indication of the diversity of each pair of the accessions). Similarly, PCoA analysis was employed using polymorphic markers from rye and triticale genome.

Model-based clustering, employing a Bayesian algorithm, was applied to infer the genetic structure of the 46 tritordeum accessions using STRUCTURE software version 2.3.1 [57]. The program was run assuming a population admixture model and correlated allele frequencies. The number of assumed groups (K) was set to vary between 1 and 10, and for each value of K five times independently MCMC (Markov Chain Monte Carlo) of 50,000 iterations was run in order to verify that the estimates were consistent across runs, of which the first 10,000 were discarded as burn-in. The likelihood of the data for a given number of assumed groups (K) is provided by the software, and the value of K with the highest likelihood can be interpreted to correspond to an estimate for the underlying number of groups. Within the admixture model we can obtain the membership probabilities of each genotype to each group inferred.

### Genomic in situ hybridization (GISH)

Root tips of 1-cm length were collected from germinating seeds and pre-treated for 4h in an aqueous colchicine solution (0.05%) at 25°C. They were fixed in a freshly prepared 3 absolute ethanol: 1 glacial acetic acid (*v*/*v*) mixture and stored at 4°C during 1 month approximately. Preparations were made as described by [58]. Total *H. chilense* genomic DNA was labelled by nick translation with biotin-11-dUTP (Roche Corporation, Basel, Switzerland) and total *Secale cereale* genomic DNA was labelled with digoxigenin-dUTP. Both probes were mixed in the hybridization solution to a final concentration of 5 ng/ml. Biotin- and digoxigenin-labelled probes were detected with streptavidin-Cy3 conjugates (Sigma, St Louis, MO, USA) and antidigoxigenin-FITC (Roche Corporate) respectively. Chromosomes were counterstained with DAPI (4′, 6-diamidino-2-phenylindole) and mounted in Vectashield (Vector Laboratories Inc.). Slides were examined by using a Zeiss LSM 5 Pa confocal laser scanning microscope with LSM 5 Pascal software version 3.0 (Zeiss, Jena, Germany).

## Competing interests

The authors declare that they have no competing interests.

## Authors’ contributions

AC performed SSR, EST and GISH genotyping, data analysis, interpretation of results and paper writing. MCR participated in chromosome counting, GISH genotyping and plant material development. ACM participated in SSR, EST and GISH genotyping. AK participated in the DArT genotyping. AM developed all the plant materials, conception, design of the study and interpretation of the results. SGA participated in design of the study, data analysis, interpretation of results and paper writing. All authors read and approved the final manuscript.

## Supplementary Material

Additional file 1 **Polymorphic markers found among 46 tritordeum accessions.***H. chilense* and D- genome markers used for genetic analysis are shown in bold. Click here for file

Additional file 2 **Markers assigned to a specific *H. chilense *chromosome using wheat-*H. chilense *addition lines.** Click here for file

Additional file 3 Markers discriminating tritordeums carrying the 1RS/1BL translocation. Click here for file
